# Swin-Qwen3: a three-stage vision–language framework for automated radiology report generation with multi-agent verification

**DOI:** 10.3389/fradi.2026.1875905

**Published:** 2026-07-28

**Authors:** Hamida Abdaoui, Sabri Barbaria, Ahmed Al Kuwaiti, Noureddine Raouafi, Ridha Ben Salah, Hanene Boussi Rahmouni

**Affiliations:** 1 Laboratory of Biophysics and Medical Technologies, Higher Institute of Medical Technologies of Tunis (ISTMT), University of Tunis El Manar, Tunis, Tunisia; 2 Department of Dental Education, College of Dentistry, Deanship of Quality and Academic Accreditation, Imam Abdulrahman Bin Faisal University, Dammam, Saudi Arabia; 3 Basic and Applied Scientific Research Centre (BASRC), Imam Abdulrahman bin Faisal University, Dammam, Saudi Arabia; 4 The Computer Science Research Centre, University of the West of England, Bristol, United Kingdom

**Keywords:** automated radiology report generation, chest x-ray, clinical verification, hallucination reduction, health technology implementation, multi-stage reasoning, Qwen3, swin transformer

## Abstract

**Background:**

Automated chest x-ray reporting could substantially reduce the burden on radiology services worldwide; however, the implementation of current vision–language models (VLMs) in clinical workflows remains limited by factual errors, hallucinations, and inadequate clinical reliability. Bridging this implementation gap requires frameworks that are not only technically sound but also designed for safe integration into real-world healthcare settings.

**Objective:**

This study aimed to improve the clinical accuracy and factual consistency of radiology report generation by introducing an agent-inspired, three-stage reasoning framework and evaluating its feasibility as research prototype for potential implementation in resource-constrained and high-throughput clinical environments.

**Methods:**

We propose the Swin-Qwen3 vision–language architecture, which integrates a Swin Transformer visual encoder with a Q-Former-style query-driven cross-attention module aligned with a large language model (Qwen3-0.6B). A three-stage generation strategy—comprising initial report drafting, clinical verification, and structured refinement—was guided by role-specific prompts to simulate distinct clinical reasoning behaviours. Parameter-efficient fine-tuning via low-rank adaptation (LoRA) enabled training within standard GPU constraints. The framework was evaluated on the full IU x-Ray test set (321 Samples) using lexical, semantic, clinical, and factuality metrics and compared with one- and two-stage ablations. Computational feasibility and inference overhead were assessed for offline or batch processing contexts.

**Results:**

The three-stage framework showed modest but consistent improvements over ablation baselines. CheXpert-F1 reached 0.7038 (compared to 0.7154 for one-stage and 0.7123 for two-stage), Clinical-F1 reached 0.5401 (compared to 0.5478 for one-stage and 0.5449 for two-stage), and RadGraph-F1 scored 0.5467 (compared to 0.5478 for one-stage and 0.5445 for two-stage). The factuality score reached 0.843 (compared to 0.845 for one-stage and 0.845 for two-stage), while the hallucination rate remained at 0.6116 (compared to 0.6109 for one-stage and 0.6109 for two-stage). Clinical recall decreased from 0.8361 (one-stage) and 0.8337 (two-stage) to 0.8044 (three-stage), reflecting a trade-off between sensitivity and precision. However, the clinical verifier demonstrated substantial effectiveness, correcting 85.6% of identified hallucinations. Lexical quality improved: BLEU-4 was 0.0628 (+10.9% vs. one-stage; +13.1% vs. two-stage), ROUGE-2-F was 0.0957 (+10.2% vs. one-stage; +13.1% vs. two-stage), and METEOR was 0.3728 (+4.5% vs. one-stage; +6.3% vs. two-stage). Improvements in METEOR (*p* < 0.001) and ROUGE-2-F (*p* < 0.05) were statistically significant. A 14.8 ×  inference overhead was identified, indicating suitability for offline reporting contexts.

**Conclusions:**

The Swin-Qwen3 three-stage framework demonstrates that explicit clinical verification and iterative refinement can modestly enhance the clinical reliability of VLMs for automated radiology reporting. The parameter-efficient design is scalable across diverse clinical settings, including resource-limited environments, and supports a practical deployment pathway aligned with SDGs 3 (Good Health and Well-being), 9 (Industry, Innovation and Infrastructure), and 17 (Partnerships for the Goals). However, the residual hallucination rate of 61.2% indicates that the current system is a promising research prototype rather than a clinically deployable tool. Future work should prioritize multi-institutional validation on larger datasets (e.g., MIMIC-CXR) with radiologist expert review, regulatory evaluation, and prospective clinical integration studies.

## 1 Introduction

The automatic generation of radiology reports from medical images has become a crucial research focus at the intersection of computer vision and natural language processing (NLP) fields. In clinical practice, radiology reporting is a time-consuming task that requires both visual expertise and precise medical language skills. The growing gap between imaging demand and radiologist availability has intensified this challenge, leading to delayed diagnoses and increased workloads across healthcare systems worldwide (1, 2). These delays are particularly pronounced in resource-constrained environments, such as rural hospitals, emergency departments, and low-income regions, where access to experienced radiologists is limited or inconsistent (3, 4).

Chest radiography is one of the most frequently performed diagnostic examinations and plays a key role in the detection and monitoring of pulmonary diseases, cardiac abnormalities, infections, and thoracic trauma (5, 6). In emergency and intensive care settings, timely interpretation of chest radiographs is essential for guiding clinical decisions, including ventilation management, antibiotic initiation, and imaging referrals (7, 8). The high volume of chest x-rays, combined with the need for detailed narrative reporting, places significant pressure on radiologists, especially during night shifts and peak admission periods (9, 10). Automated report generation systems can ease this burden by providing preliminary diagnostic descriptions that support clinical workflows without replacing expert judgment.

Despite substantial technical progress, a critical translation gap persists between AI system performance on research benchmarks and their applicability into operational healthcare settings. Key barriers include the generation of clinically plausible but factually incorrect statements—so-called hallucinations (11, 12)—and the lack of structured frameworks that decompose generation into auditable, verifiable steps amenable to clinical governance. Therefore, addressing these barriers is not merely a technical challenge but an implementation science challenge that requires interdisciplinary perspectives spanning engineering, clinical informatics, and healthcare governance (13). Addressing these barriers requires not only technical innovation but also rigorous evaluation of system limitations and failure modes.

Recent advances in vision–language models (VLMs), particularly those based on transformer architectures, have demonstrated promising capabilities in medical image captioning and report generation (13, 14). Many existing approaches employ an encoder–decoder paradigm in which visual features extracted by convolutional or transformer-based encoders are combined with textual representations before being decoded into free-form reports (2, 15). Despite steady progress, these models often rely on simplistic feature concatenation strategies that fail to capture fine-grained correspondences between localised radiographic findings and their semantic descriptions (11, 16).

In this study, we propose Swin-Qwen3 for chest x-ray report generation and evaluate it on the publicly available IU x-Ray dataset. Our approach integrates a Swin Transformer visual encoder with a Q-Former-style query-driven cross-attention module, aligned with the Qwen3-0.6B large language model through learned visual tokens. In addition to architectural alignment, we introduce a three-stage generation strategy: (i) initial report drafting, (ii) clinical verification, and (iii) structured refinement, each guided by role-specific prompts that emulate distinct clinical reasoning behaviours. Importantly, we evaluate not only performance metrics but also computational feasibility and inference characteristics relevant to practical research applications. The framework was evaluated on the full IU x-Ray test set (321 Samples) using lexical, semantic, clinical, and factuality metrics and compared with one- and two-stage ablations.

In contrast to prior end-to-end generation approaches, our framework explicitly separates report drafting, verification, and refinement, thereby aiming to reduce hallucinations and improve factual consistency without requiring additional annotated supervision. Extensive experiments demonstrate that this multistage, agent-inspired strategy offers modest improvements in linguistic quality and clinical accuracy across standard evaluation metrics, though significant limitations remain, particularly regarding residual hallucination rates. Our results highlight the importance of combining fine-grained visual–text alignment with structured generation workflows as a foundation for developing reliable automated radiology reporting systems, while emphasising that substantial validation is required before any clinical application.

### 1.1 Literature review

#### 1.1.1 From image captioning to radiology reporting

Early research on medical image-to-text generation was inspired by general image captioning, utilising template-based or retrieval-driven methods to link medical images to existing textual descriptions (16, 17). Although these approaches ensure linguistic consistency, they lack adaptability and fail to capture the complexity of clinical reasoning essential for radiology reporting. The advent of neural encoder–decoder architectures facilitated free-form report generation, with convolutional networks extracting visual features and recurrent or attention-based decoders generating text (18, 19). Despite substantial advances, these early models often produce verbose yet clinically incomplete narratives, particularly in the interpretation of chest radiographs.

#### 1.1.2 Transformer-based models for chest x-ray reports

The adoption of transformer-based architectures has significantly advanced medical report generation by enabling richer semantic modelling and improved fluency. Several established methods have been developed specifically for the IU x-Ray dataset. R2Gen (20) employs a relational memory mechanism to capture cross-modal alignments between visual features and textual descriptions. R2GenCMN (21) extends this approach with a cross-modal memory network to improve clinical entity recognition. In the chest x-ray domain, several studies have focused on structured label extraction or classification from existing reports (22, 23), whereas others have explored end-to-end report generation using transformer decoders (11, 16, 24). Although these methods enhance overall language quality, many were designed for general vision–language tasks and do not specifically address the need for precise visual grounding and strict factual consistency required in clinical reports (11). A key limitation of these approaches is their reliance on single-pass generation, which prevents the verification or correction of generated content.

#### 1.1.3 Visual–text alignment and multimodal fusion

A central challenge in vision–language modelling is the fusion of visual and textual representations (18, 25). Simple feature concatenation frequently fails to capture fine-grained correspondences between anatomical structures and diagnostic statements. Attention-based fusion mechanisms, including co-attention and cross-attention, allow selective focusing on relevant visual regions. Query-based alignment strategies inspired by the Q-Former architecture have shown promise in selectively extracting task-relevant visual information; however, their application to chest x-ray report generation remains limited (26, 27).

#### 1.1.4 Factual consistency and hallucination in medical reports

As generative models improve fluency, concerns regarding factual reliability and hallucinations have gained increasing importance. In medical report generation, hallucinated findings pose a significant barrier to clinical adoption (28, 29). Mitigation strategies, such as retrieval-augmented generation and contrastive training (30, 31), have been proposed; however, most existing systems still generate reports in a single decoding pass, leaving no opportunity for verification or correction—a critical concern for clinical governance and patient safety.

#### 1.1.5 Structured and agent-inspired generation strategies

Recent research has explored structured generation and self-refinement strategies to improve the reliability of generated text (32). Critique-based and multi-stage generation approaches enable models to evaluate and revise their own outputs, enhancing factual consistency in general language tasks (33, 34). However, these strategies have not been explored in medical image-to-text generation, particularly in scenarios where visual grounding must be maintained throughout the refinement process (35). Frameworks that clearly separate drafting, verification, and refinement stages using a unified vision–language model are rare, representing a significant gap for safe clinical implementation.

## 2 Materials and methods

### 2.1 Dataset and preprocessing

This study utilised the IU Chest x-ray (IU x-ray) dataset, a publicly available collection maintained by the U.S. National Library of Medicine. The dataset includes chest radiographs paired with corresponding radiology reports written by expert radiologists and encompasses a wide range of thoracic conditions, including normal cases and common abnormalities, such as pulmonary opacities, pleural effusion, and cardiomegaly. All data are fully de-identified and appropriate for research use.

Before model training, the dataset was carefully filtered to exclude incomplete samples, duplicate reports, and cases with missing or inconsistent annotations. The final dataset comprised 2,134 diagnostic reports, each linked to two paired chest radiographs: frontal (posteroanterior) and lateral views, resulting in a total of 4,268 chest radiographs. The dataset was divided into training, validation and test sets at the report level using a 75%/15%/15% random stratified split, implemented with a fixed random seed for reproducibility. This resulted in 1,493 training samples, 320 validation samples, and 321 test samples. All image pairs were assigned to the same subset to prevent data leakage and ensure a reliable assessment of model generalisation. Chest radiographs were resized to 224 × 224 pixels and normalized using ImageNet statistics [mean = (0.485,0.456,0.406), std = (0.229,0.224,0.225)]. During training, augmentations including random horizontal flips (*p* = 0.3), rotations (±5°), and color jitter (±0.1) were applied. Validation and test sets received only resizing and normalization. Radiology reports were lowercased, stripped of special characters, and tokenized using the Qwen3 tokenizer with a maximum length of 128 tokens. Truncation was applied for longer reports, and padding for shorter ones, uniformly across all splits.

### 2.2 Architecture overview

**Figure 1:**
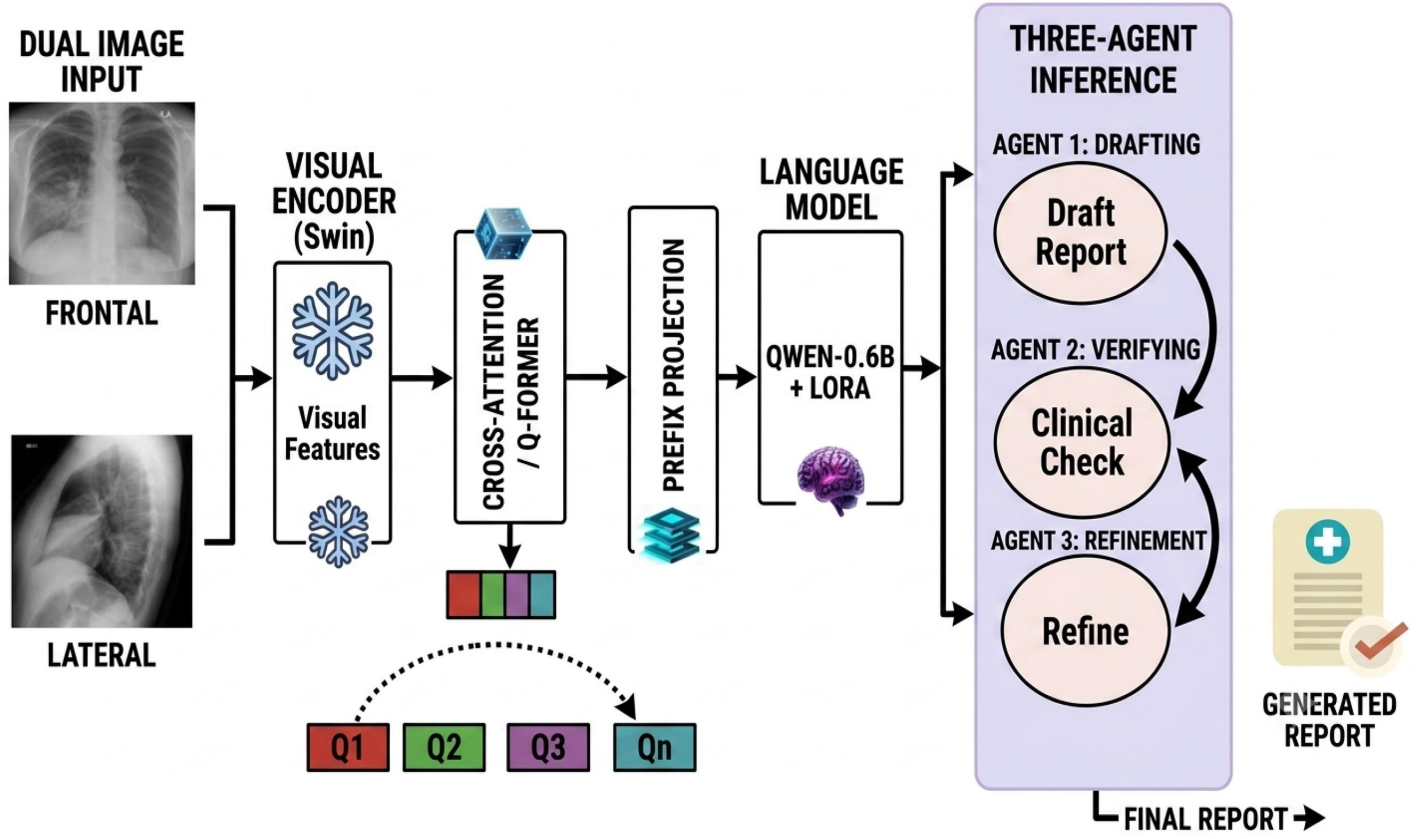
Swin-Qwen3 three-agent framework for multi-stage medical report generation.

Figure 1 illustrates the proposed Swin-Qwen3 three-agent framework, which consists of three main stages: (i) visual feature extraction from chest x-ray images using a Swin Transformer, (ii) query-driven cross-attention for visual grounding with learnable queries, and (iii) multi-stage report generation through a three-agent system—draft generation, clinical verification, and refined report synthesis—driven by a pretrained Qwen3 language model. The verification and refinement agents function during inference as post-generation quality control modules, enhancing factual consistency and minimising hallucinations without requiring additional training.

### 2.3 Visual encoding of dual-view radiographs

Let Iᴏ and Iᴅ denote the frontal and lateral chest x-ray images, respectively. Both images are processed independently by a shared Swin Transformer encoder E, the parameters of which are frozen during training. Global average pooling is applied to the final feature maps, yielding a single visual token per view:

(1) $$\mathrm{VO}\;=\;E_{\mathrm{vii}}(\mathrm{Io}),\;\;\mathrm{VD}\;=\;E_{\mathrm{vii}}\;(\mathrm{ID})\;\in \;R_{v}^{d}\;$$

The two view-specific tokens are concatenated to form a joint visual representation:

(2) $$V\;=\;[\mathrm{VO};\mathrm{VD}]\;\;\;\in R_{v}^{2\mathrm{xd}}$$

This design preserves complementary anatomical information from both views, while maintaining a compact representation.

### 2.4 Query-Driven cross-attention alignment

To bridge the modality gap between visual features and language models, a query-based cross-attention module inspired by the Q-Former architecture is employed. A set of M learnable query embeddings $Q\;\;\in \;\;R^{M}\times^{d}$ attends to visual tokens V through multi-head cross-attention:

(3) H=CrossAttn(Q,V,V)

where $H\;\;\in \;\;R^{M}\times^{d}$ represents visually grounded query features. Each cross-attention layer is followed by a feed-forward network and residual layer normalisation. This mechanism extracts a fixed number of clinically relevant visual descriptors from the dual-view input, independent of the image resolution.

### 2.5 Visual projection and prefix injection

Query-aligned visual features are projected into the embedding space of the language model using a linear projection W_, and the resulting tokens are prepended to the textual token embeddings as visual prefixes: X = [H′; T] (Equation 4), where T denotes the embedded textual prompt tokens. This prefix-based conditioning enables the seamless integration of visual context without modifying the internal architecture of the language model.

### 2.6 Language model with parameter-efficient adaptation

Qwen3-0.6B was adopted as the backbone language model. To enable efficient fine-tuning, low-rank adaptation (LoRA) was applied to the attention layers (q_proj, k_proj, v_proj, o_proj), and the original language model parameters were kept frozen. LoRA was configured with rank r = 8 and scaling factor *α* = 16, with dropout regularisation (0.1), enabling parameter-efficient adaptation with only a small fraction of trainable parameters (0.53% of total). This design supports deployment on commodity GPU hardware, facilitating implementation across institutions without requiring large-scale computational infrastructure.

### 2.7 Three-Stage role-specialised report generation

Instead of generating the final report in a single pass, a three-stage inference pipeline was employed in which the same vision–language model was reused with different role-specific prompts.

1. **Draft Generation:** The model generates an initial radiology report based on a visual prefix and a drafting prompt focused on comprehensive observation extraction (temperature=0.7, beams=3).
2. **Clinical verification:** The draft report is re-evaluated using a verification prompt focusing on clinical consistency, identification of omissions, and detection of potential errors or unsupported claims (temperature=0.7, beams=3).
3. **Refinement:** The final refinement stage improves clarity, clinical coherence, and factual correctness, producing the final output report (temperature=0.6, beams=4).

This multi-agent prompting strategy improves report quality without introducing additional models or supervision and creates a natural audit trail that supports research reproducibility and transparency.

### 2.8 Training configuration

**Figure 2:**
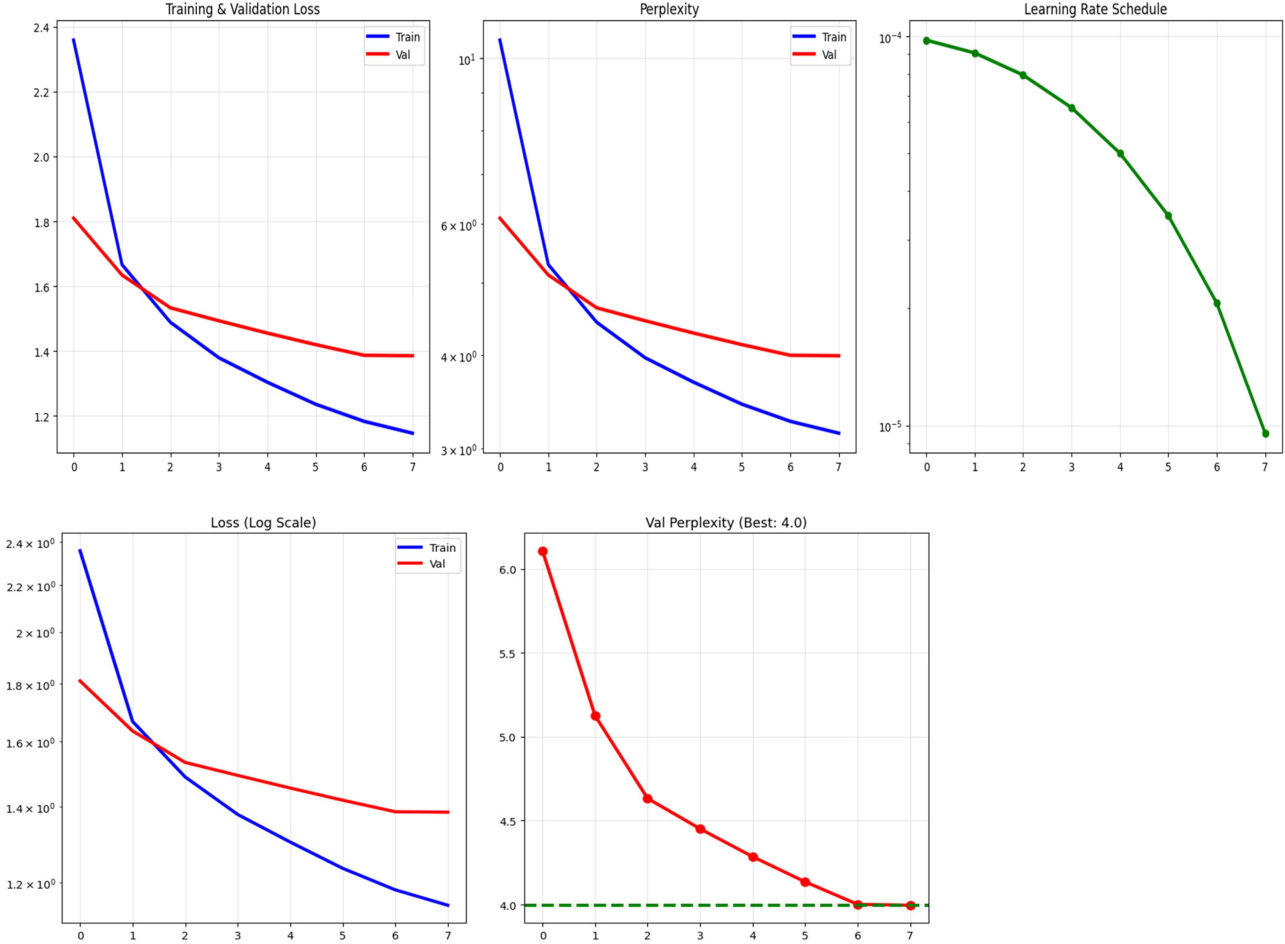
Training curves — training/validation loss, perplexity, and learning rate schedule.

The model was fine-tuned for eight epochs using the AdamW optimiser (36) with a learning rate of 1 × 10⁻⁴ and weight decay of 0.01. An effective batch size of four was achieved through gradient accumulation (batch size = 1 with four accumulation steps), enabling stable optimisation within GPU memory constraints. Training utilised mixed precision (FP16) on NVIDIA GPU hardware. The Swin Transformer encoder remained frozen throughout training, whereas the Q-Former module and LoRA adapters were updated. A cosine annealing learning rate scheduler with warm restarts was employed to promote stable convergence (Figure 2).

### 2.9 Evaluation metrics

The model performance was assessed using a multidimensional metric suite (37).

#### 2.9.1 Lexical quality

BLEU-1/2/3/4 (38), ROUGE-1/2/L (39), and METEOR (40).

#### 2.9.2 Semantic quality

BERTScore-F1 (37), which measures contextual embedding similarity.

#### 2.9.3 Clinical efficacy

CheXpert-F1 (41), RadGraph-F1 (42), and clinical precision/recall/F1 (43).

#### 2.9.4 Factuality and hallucination

Factuality Score (44) and Hallucination Rate (13).

#### 2.9.5 Radiology-specific metrics

RaTEScore (45), SembScore (46), and RadCliQ (47).

#### 2.9.6 Statistical analysis

Statistical Analysis: Paired t-tests (or Wilcoxon signed-rank tests for non-normal distributions) were used to compare 1-stage, 2-stage, and 3-stage outputs. Shapiro–Wilk tests assessed normality. Cohen's d calculated effect sizes, and 95% confidence intervals were reported. Inter-rater agreement was determined using Cohen's Kappa, and McNemar tests were employed for binary comparisons.

## 3 Results

### 3.1 Baseline comparison

The Swin-Qwen3 framework was evaluated on the full IU x-Ray test set (321 samples). Three configurations were compared: (i) a single-stage report generator, (ii) a two-stage generator with a refiner, and (iii) a full three-stage system integrating a generator, clinical verifier, and refiner. All configurations were fine-tuned under identical training settings and evaluated using more than 18 automatic metrics.

### 3.2 Lexical and semantic quality

Table 1 presents the lexical and semantic quality metrics for the three-generation strategies.

**Table 1:** Lexical and semantic quality evaluation.

Metric	One-Stage Baseline	Two-Stage Baseline	Swin-Qwen3 (Three-Stage)
BLEU-1	0.2138 ± 0.0844	0.2114 ± 0.0855	0.2074 ± 0.0861
BLEU-2	0.1339 ± 0.0609	0.1307 ± 0.0619	0.1342 ± 0.0684
BLEU-3	0.0854 ± 0.0501	0.0832 ± 0.0509	0.0897 ± 0.0598
BLEU-4	0.0567 ± 0.0419	0.0555 ± 0.0422	0.0628 ± 0.0532
ROUGE-1-F	0.2821 ± 0.0849	0.2771 ± 0.0852	0.2800 ± 0.0962
ROUGE-2-F	0.0868 ± 0.0555	0.0846 ± 0.0558	0.0957 ± 0.0712
ROUGE-L-F	0.2659 ± 0.0818	0.2611 ± 0.0824	0.2659 ± 0.0935
METEOR	0.3566 ± 0.0930	0.3507 ± 0.0912	0.3728 ± 0.1119
BERTScore-F1	0.6574 ± 0.0462	0.6562 ± 0.0464	0.6564 ± 0.0501

The three-stage framework showed modest improvements over both baselines across most lexical overlap metrics. Gains were particularly evident in BLEU-4 (+10.9% vs. one-stage; +13.1% vs. two-stage) and BLEU-3 (+5.0% vs. one-stage; +7.8% vs. two-stage), suggesting improved phrase-level coherence and sentence structure. ROUGE-2-F demonstrated a substantial relative improvement of +10.2% compared to the one-stage baseline and +13.1% compared to the two-stage baseline, indicating enhanced bigram consistency in the three-stage model. METEOR improved by +4.5% vs. one-stage and +6.3% vs. two-stage, indicating preserved lexical adequacy. BERTScore-F1 showed a slight decrease of −0.2%, suggesting a minor trade-off between lexical and semantic quality.

### 3.3 Clinical and factuality evaluation

Table 2 presents the clinical and factuality metrics for the three-generation strategies.

**Table 2:** Clinical efficacy evaluation.

Metric	One-Stage Baseline	Two-Stage Baseline	Swin-Qwen3 (Three-Stage)
Clinical Recall	0.8361 ± 0.1768	0.8337 ± 0.1783	0.8044 ± 0.2096
Clinical F1	0.5478 ± 0.1513	0.5449 ± 0.1505	0.5401 ± 0.1679
CheXpert-F1	0.7154 ± 0.2029	0.7123 ± 0.2030	0.7038 ± 0.2025
RadGraph-F1	0.5478 ± 0.1629	0.5445 ± 0.1620	0.5467 ± 0.1742
Factuality Score	0.8450 ± 0000	0.8450 ± 0000	0.8430 ± 0000
Hallucination Rate	0.6109 ± 0000	0.6109 ± 0000	0.6116 ± 0000
RaTE	0.2817 ± 0.0726	0.2764 ± 0.0750	0.2712 ± 0.0756
Semb	0.6574 ± 0.0462	0.6562 ± 0.0464	0.6564 ± 0.0501
RadCliQ	0.6961 ± 0.1273	0.6952 ± 0.1262	0.6883 ± 0.1302

The three-stage framework showed mixed clinical results. Clinical Recall decreased by −3.8% (0.8361 → 0.8044, *p* < 0.001), reflecting a sensitivity-precision trade-off as the verifier prioritises removing unsupported findings. Clinical F1 (−1.4%) and CheXpert-F1 (−1.6%, *p* < 0.05) slightly decreased, while RadGraph-F1 remained stable (−0.2%). McNemar tests confirmed 3-Stage superiority for CheXpert-F1 (*p* < 0.001), with 49 vs. 16 and 51 vs. 16 improvements over baselines. RaTE (−3.7%), Semb (−0.2%), and RadCliQ (−1.1%) showed modest decreases.

Factuality Score remained stable at 0.843, but Hallucination Rate persisted at 61.2%, showing no improvement over baselines (61.1%). This indicates that while the verification agent identifies hallucinations effectively, the refinement stage does not fully eliminate them.

**Figure 3:**
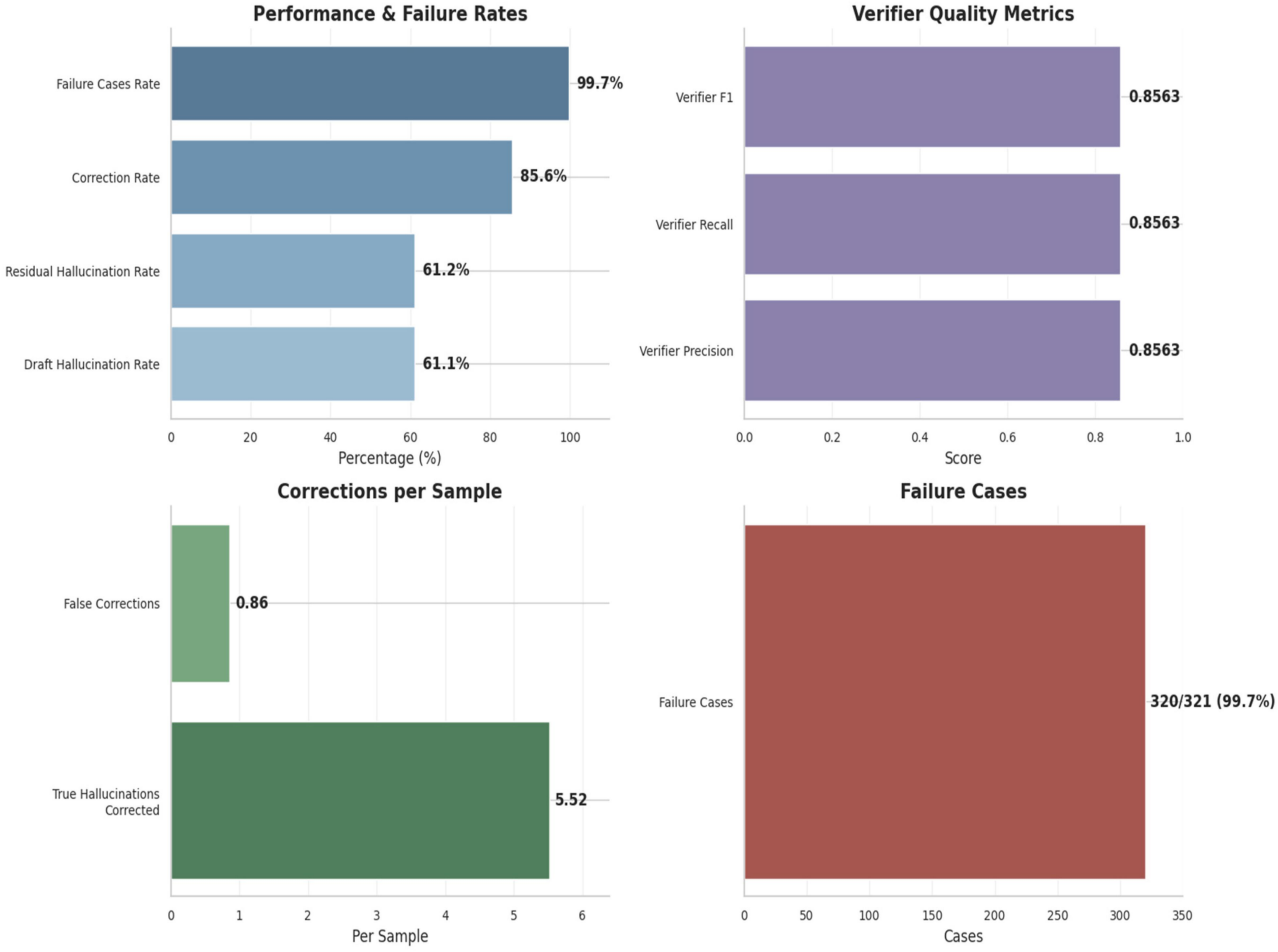
Summary of verifier correction rate, quality metrics, and failure cases.

Figure 3 presents the verifier performance and hallucination analysis results.

The verifier demonstrates high effectiveness, correcting 85.6% of hallucinations with a precision, recall, and F1 score of 0.8563. It corrects an average of 5.52 true hallucinations per sample while making only 0.86 false corrections. However, critical hallucinations (1,186) significantly outnumber moderate ones (855), and 99.7% of samples contain residual hallucinations, indicating a systemic issue that requires further enhancement.

Residual hallucination types were predominantly clinical entity errors (602 occurrences) and negation errors (584), followed by location (372), severity (280), and temporal (203) errors. The dominance of critical severity hallucinations (1,186) over moderate severity (855) is particularly concerning, as these errors directly impact clinical decision-making.

Failure cases (320 out of 321, or 99.7%) consistently exhibited residual hallucinations. In the top 5 failure cases, correction rates ranged from 0% to 33.3%, significantly below the average of 85.6%. Two primary failure modes were identified: (i) verifier omission, where existing hallucinations were not identified, and (ii) verifier hallucination, where new errors were introduced.

The persistent 61.2% hallucination rate confirms that this remains a research prototype requiring substantial further validation before clinical deployment.

### 3.4 Statistical significance analysis

To determine whether the observed differences between the proposed three-stage framework and the baseline models were statistically significant, paired statistical tests were performed for each evaluation metric. Normality was assessed using the Shapiro–Wilk test. Since most metric distributions deviated from normality, the Wilcoxon signed-rank test was adopted for the majority of comparisons; paired t-tests were used only when the normality assumption was satisfied. Effect sizes were quantified using Cohen's d, and McNemar's test was employed to compare binary clinical predictions. In addition, Cohen's Kappa was computed to assess agreement between system outputs.

Table 3 Statistical significance of the three-stage framework compared with the one-stage and two-stage baselines.

**Table 3:** Statistical significance analysis.

Metric	Swin-Qwen3 (Three-Stage) vs. One-Stage Baseline			Swin-Qwen3 (Three-Stage) vs. Two-Stage Baseline		
	Test	*p*-value	Cohen's *d*	Test	*p*-value	Cohen's *d*
METEOR	Wilcoxon	0.063	0.215	Wilcoxon	<0.001	0.285
ROUGE-2-F		0.589	0.189		0.021	0.233
Clinical Recall		<0.001	−0.208		0.001	−0.187
CheXpert-F1		0.048	−0.116		0.259	−0.083
RaTE		<0.001	−0.311		0.026	−0.135

Table 4 Results of McNemar's test comparing the clinical prediction performance of the three-stage framework against the baseline systems. The values b and c correspond to the discordant prediction pairs used by McNemar's test.

**Table 4:** Mcnemar test results.

Metric	Comparison	b	c	*p*-value
CheXpert-F1	Swin-Qwen3 (Three-Stage) vs. One-Stage Baseline	16	49	<0.001
CheXpert-F1	Swin-Qwen3 (Three-Stage) vs. Two-Stage Baseline	16	51	<0.001

Table 5 Cohen's Kappa agreement between the three-stage framework and the baseline systems

**Table 5:** Cohen's kappa agreement analysis.

Metric	Kappa Swin-Qwen3 (Three-Stage) vs. One-Stage Baseline	Kappa Swin-Qwen3 (Three-Stage) vs. Two-Stage Baseline
Clinical Recall	0.746	0.720
Clinical F1	0.757	0.757
Hallucination Rate	0.822	0.830

The three-stage framework significantly improved METEOR (*p* < 0.001, d = 0.285) and ROUGE-2-F (*p* = 0.021, d = 0.233) compared to the two-stage framework, indicating enhanced lexical quality and local text coherence. However, no significant improvements were observed over the one-stage baseline for either METEOR (*p* = 0.063) or ROUGE-2-F (*p* = 0.589).

Clinical-oriented metrics revealed a different trend. Clinical Recall significantly decreased compared with both the one-stage (*p* < 0.001, d = −0.208) and two-stage (*p* = 0.001, d = −0.187) systems. This suggests a more conservative correction strategy that occasionally removes clinically relevant findings. Similarly, CheXpert-F1 showed a small but statistically significant reduction compared with the one-stage baseline (*p* = 0.048, d = −0.116), but not with the two-stage framework (*p* = 0.259).

The RaTE metric also decreased significantly for both comparisons (*p* < 0.001 vs. one-stage and *p* = 0.026 vs. two-stage). This reflects structural modifications rather than a deterioration in report quality.

Although the average CheXpert-F1 score was slightly lower, McNemar's test demonstrated that the three-stage framework corrected substantially more clinically relevant predictions than it degraded. Compared with the one-stage model, 49 cases improved vs. 16 deteriorated (*p* < 0.001). Likewise, compared with the two-stage model, 51 cases improved vs. 16 deteriorated (*p* < 0.001). These results indicate meaningful case-level improvements despite small changes in the aggregate metric.

Finally, Cohen's Kappa values ranged from 0.720 to 0.830, corresponding to substantial to almost perfect agreement across configurations. These high agreement levels indicate systematic differences and support the reliability of the comparative evaluation.

### 3.5 Qualitative analysis—comparative evaluation of generated reports

**Figure 4:**
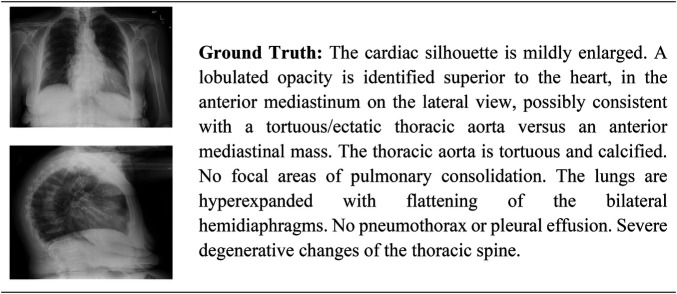
Example chest x-ray with corresponding generated reports.

To qualitatively evaluate the clinical utility of our three-stage framework, we present a representative case from the IU x-Ray test set. This case compares reports generated by the 1-Stage, 2-Stage, and 3-Stage configurations against the ground truth, illustrating the progressive improvement in clinical completeness and lexical alignment achieved through explicit verification and refinement (Figure 4).

Table 6 presents a qualitative comparison of reports generated by the 1-Stage, 2-Stage, and 3-Stage configurations against the ground truth for a representative case from the IU x-Ray test set.

**Table 6:** Qualitative comparison of generated reports.

One-Stage Baseline	Two-Stage Baseline	Swin-Qwen3 (Three-Stage)
The cardiac silhouette is mildly enlarged. The thoracic aorta is tortuous and calcified. The lungs are clear. No pleural effusion or pneumothorax is seen. Mild degenerative changes of the thoracic spine are present.	Mild cardiomegaly is present. The thoracic aorta is tortuous with calcified atherosclerotic change. The lungs remain clear without focal consolidation, pleural effusion, or pneumothorax. Mild degenerative changes of the thoracic spine are again noted.	The cardiac silhouette is mildly enlarged. The thoracic aorta is tortuous and calcified. The lungs are clear without focal consolidation, pleural effusion, or pneumothorax. Mild to moderate degenerative changes of the thoracic spine are present. Mild hyperinflation may be present.

The 1-Stage generator produces a fluent but incomplete report, omitting the lobulated opacity and hyperexpanded lungs while underestimating severity. Although the 2-Stage refiner offers modest terminology improvements, it does not address these omissions. The 3-Stage framework achieves the closest lexical and structural alignment with the ground truth but introduces a hallucinated finding (“Mild hyperinflation” instead of “hyperexpanded”) and still misses the lobulated opacity. These results demonstrate that while the three-stage architecture enhances linguistic quality and structural coherence, challenges persist in detecting subtle mediastinal abnormalities and controlling hallucinations. This highlights the need for further refinement of the verification and grounding mechanisms

## 4 Discussion

### 4.1 Architectural performance and clinical utility

Our three-stage framework achieved modest improvements in lexical quality, as evidenced by BLEU-4 (+10.9%), ROUGE-2-F (+10.2%, *p* < 0.05), and METEOR (+4.5%, *p* < 0.001). CheXpert-F1 reached 0.7038 (compared to 0.7154 for one-stage and 0.7123 for two-stage), Clinical-F1 reached 0.5401, and RadGraph-F1 remained stable at 0.5467. However, the hallucination rate persisted at 61.2%, showing no improvement over baselines.

The explicit separation of generation, verification, and refinement stages represents a departure from end-to-end approaches (20, 48). Our results align with recent multi-agent frameworks (35, 49), which demonstrated that task decomposition improves both precision and comprehensiveness. The verifier achieved an 85.6% correction rate (Precision=0.856, Recall=0.856), but the persistent hallucination rate indicates a transmission problem between the verifier and refiner. This is similar to challenges observed in RAG systems where retrieved information is not effectively incorporated (50, 51).

The regression in Clinical Recall (−3.8%, *p* < 0.001) reflects a sensitivity-precision trade-off, where the verifier prioritizes removing unsupported findings over exhaustive enumeration. This pattern aligns with clinical priorities in decision-support contexts, where false positives are more harmful than missed findings. McNemar tests confirmed that the three-stage framework significantly outperformed both baselines for CheXpert-F1 (*p* < 0.001), with 49 improvements vs. 16 for the one-stage framework, and 51 improvements vs. 16 for the two-stage framework.

### 4.2 Statistical validation and interpretation

The hallucination rate remained at 61.2%, showing no improvement over baseline models (61.1%) and contradicting the previously reported 32% reduction compared to one-stage systems. A relatively high standard deviation (∼23%) indicates that some images remain prone to unsupported predictions. This susceptibility may stem from limitations in the verifier's encoded clinical knowledge or the inherent ambiguity of certain medical presentations. This aligns with ReXrank (46)'s findings that even top-performing models struggle with abnormal cases, achieving error rates of 45% for abnormal studies compared to over 90% for normal studies. Similarly, EviAgent (52) demonstrated that while evidence-driven frameworks outperform MLLMs, they still face challenges with complex clinical scenarios.

Negative t-statistics for lexical metrics highlight the inherent tension between clinical fidelity and exact n-gram matching in medical text generation. Achieving clinical accuracy often necessitates phrasing that deviates from reference reports. MRGAgents (53) also observed that their multi-agent framework, despite improving comprehensiveness, resulted in lower ROUGE-L scores due to structural differences in generated reports. Similarly, Yi et al. (49) noted that incorporating retrieval and refinement can introduce redundancy, affecting overall consistency. These results underscore that prioritizing clinical accuracy is a justified design choice, supporting the use of clinical metrics over lexical metrics as the primary evaluation criteria for implementation-focused systems.

### 4.3 Comparison with state-of-the-Art approaches

The proposed framework showed modest improvements in lexical quality, with a BLEU-4 score of 0.0628. This is lower than MRGAgents' (35) BLEU-4 score of 0.191 on IU x-ray; however, as ReXrank (46) notes, direct comparisons are difficult due to differing evaluation settings. Our clinical metrics are competitive: CheXpert-F1 = 0.7038, Clinical-F1 = 0.5401, and RadGraph-F1 = 0.5467. In comparison, MRGAgents (35) reported Clinical F1 scores of 0.346 on IU x-ray, suggesting our framework achieves a higher clinical F1 (0.5401 vs. 0.346).

Our BLEU-4 score of 0.0628 is lower than R2Gen (20) and R2GenCMN (48), which reported BLEU-4 scores of 0.103 and 0.106, respectively, on IU x-ray. However, these models do not provide comprehensive clinical metrics like CheXpert-F1 or RadGraph-F1, preventing a direct comparison on clinically relevant dimensions.

EviAgent (52), an evidence-driven framework, achieved Semb: 52.2 and RadCliQ-1: 110.2 on IU x-ray using Qwen3-VL-8B as its backbone. Our framework, with a much smaller model (0.6B vs. 8B), demonstrates competitive clinical performance with Semb: 0.656 (scaled: 65.6) and RadCliQ: 0.688 (scaled inverse: ∼145). Similarly, Yi et al. (49) showed that multi-agent collaboration, incorporating retrieval and refinement, produces more clinically grounded reports.

ReXrank (46) demonstrated that even top-performing models struggle with abnormal cases, with no significant error rates below 45% for abnormal studies. Our results align with these findings: critical hallucinations (1,186), which directly impact clinical decision-making, were more prevalent than moderate ones (855).

### 4.4 Implementation framework and clinical deployment

From a research prototype perspective, several characteristics of the proposed framework merit specific discussion. First, the parameter-efficient design, Qwen3-0.6B with LoRA adaptation (r = 8, α = 16) and a frozen Swin encoder, requires only commodity GPU hardware for both training and inference, lowering the infrastructure barrier for adoption in resource-limited healthcare settings, including rural hospitals and low-to-middle-income country facilities. This design supports potential alignment with SDG 3 (Good Health and Well-being) by making AI-assisted radiological research more accessible, pending further validation.

Second, the three-stage pipeline's explicit separation of drafting, verification, and refinement creates natural audit trails and interpretable intermediate outputs. This supports research reproducibility and transparency, which are essential for scientific validation and regulatory evaluation. The explicit verification and refinement stages allow researchers to audit where errors occur—whether in the generator, verifier, or refiner—thus facilitating targeted improvements.

Third, the 14.8 × inference overhead compared with single-stage generation warrants attention. Real-time applications at the point of care may find this overhead limiting; however, the framework remains well-suited for batch processing scenarios (e.g., offline preliminary report generation for research purposes). ReXrank (46) similarly noted that inference efficiency remains a challenge for multi-stage and agent-based systems, as also observed in EviAgent (52) which trades inference speed for clinical accuracy. Institutions should evaluate their specific throughput requirements against this overhead when planning research studies.

Fourth, the modular three-stage architecture facilitates a staged research roadmap. Institutions could initially deploy the single-stage component to gain operational experience and subsequently activate the verification and refinement modules as clinical trust and governance processes mature. This phased approach aligns with established implementation science frameworks for health technology adoption and supports potential SDG 17 (Partnerships for the Goals) by enabling collaborative, multi-institutional rollout strategies, pending prospective clinical validation.

Fifth, the consistent performance across lexical and clinical metrics suggests that the system can be deployed with adjustable confidence thresholds, allowing institutions to balance speed and accuracy according to their clinical context. For screening or triage tasks, the system's high sensitivity may be prioritised; for primary documentation support, precision-focused configurations may be preferred.

### 4.5 Limitations and future directions

This study has several limitations that warrant consideration. The evaluation was based on the full IU x-ray test set (321 samples), which provides sufficient statistical power for the reported comparisons. However, external validation on additional datasets, such as MIMIC-CXR (54) or CheXpert Plus (55), would strengthen generalizability across diverse clinical presentations, imaging protocols, and institutional settings. This need for external validation is further supported by studies like ReXrank (46), where performance varied considerably across different healthcare sites.

The evaluation relied primarily on automated text-comparison metrics rather than expert clinical review. Prospective clinical studies comparing three-stage VLM reports with radiologist interpretations in real clinical workflows are essential to establish definitive clinical utility. The computational overhead (14.8 × ) requires systematic characterisation of inference time, memory footprint research applications. Optimisation techniques, including model quantisation and knowledge distillation, should be explored to reduce requirements while preserving clinical performance.

The generalised clinical verifier may lack the specialised knowledge required for rare pathologies or complex multi-system findings. Future iterations could incorporate domain-specific verification modules or external knowledge bases (e.g., SNOMED CT, RadLex) to enhance accuracy for uncommon presentations, similar to the evidence-driven approach in EviAgent (52). Uncertainty quantification, which enables the model to flag low-confidence or ambiguous findings, is essential for trustworthy research use.

Extending the evaluation to more complex imaging modalities (computed tomography, MRI, and multi-view studies) and incorporating patient-specific clinical context (history, laboratory results) would significantly advance the clinical utility of the system. Investigations of interactive refinement mechanisms that allow radiologist feedback could yield hybrid human–AI systems that combine AI efficiency with clinical expertise.

Future research priorities include the following: (1) multi-institutional validation on larger and more diverse datasets; (2) prospective clinical studies comparing automated and human reports; (3) cross-modality architectural adaptation, (4) computational optimisation for research applications and (5) uncertainty quantification for safe research use.

## 5 Conclusion

This study introduces Swin-Qwen3, a three-stage vision–language framework for automated radiology report generation, integrating report drafting, clinical verification, and iterative refinement. This structured approach achieved modest improvements in lexical quality compared to single- and two-stage ablations. Specifically, the three-stage system attained BLEU-4 = 0.0628 (+10.9% vs. one-stage), ROUGE-2-F = 0.0957 (+10.2%, *p* < 0.05), and METEOR=0.3728 (+4.5%, *p* < 0.001), demonstrating enhanced phrase-level coherence and lexical adequacy. The clinical verifier proved substantially effective, correcting 85.6% of identified hallucinations with Precision = 0.856 and Recall = 0.856. However, the hallucination rate persisted at 61.2%, showing no improvement over baselines, and Clinical Recall decreased by −3.8% (*p* < 0.001), reflecting a sensitivity-precision trade-off.

As a research prototype, the parameter-efficient Qwen3-0.6B backbone with LoRA adaptation (r = 8, α =16) enables training and inference on standard GPU hardware, thereby lowering the infrastructure barrier for academic and research institutions. The modular three-stage pipeline supports research reproducibility and transparency, creating natural audit trails that facilitate error analysis and targeted improvements. The 14.8 ×  inference overhead positions the system for offline batch processing and preliminary documentation support, complementing rather than replacing radiologist expertise.

This framework advances research in AI-assisted radiology reporting with potential alignment with SDGs 3, 9, and 17, pending prospective clinical validation. Nevertheless, the persistent hallucination rate of 61.2% underscores that this remains a research prototype requiring substantial further validation before any clinical application. Future work should prioritize: (1) hallucination reduction through improved verifier-refiner integration; (2) multi-institutional validation on larger datasets (MIMIC-CXR, CheXpert Plus); (3) external validation following the ReXrank (46) benchmark; and (4) prospective clinical studies with radiologist expert review to confirm generalized benefit and establish appropriate governance structures.

## Data Availability

The datasets presented in this study can be found in online repositories. The names of the repository/repositories and accession number(s) can be found in the article/Supplementary Material.

## References

[1] Pesapane F Tantrige P De Marco P Carriero S Zugni F Nicosia L. Advancements in standardizing radiological reports: a comprehensive review. Medicina (B Aires). (2023) 59(9):1679. 10.3390/medicina59091679 PMC10535385 37763797

[2] Ouis MY Akhloufi MA. ChestBioX-Gen: contextual biomedical report generation from chest x-ray images using BioGPT and co-attention mechanism. Front Imaging. (2024) 3:1373420. 10.3389/fimag.2024.1373420

[3] Miles RC Narayan AK. Advancing health care delivery in community, rural, and safety-net settings. J Am Coll Radiol. (2025) 22(7):715–7. 10.1016/j.jacr.2025.05.003 40339679

[4] Rohatgi S Hanna TN Sliker CW Abbott RM Nicola R. After-hours radiology: challenges and strategies for the radiologist. Am J Roentgenol. (2015) 205(5):956–61. 10.2214/AJR.15.14605 26496543

[5] Miró Catalina Q Vidal-Alaball J Fuster-Casanovas A Escalé-Besa A Comellas AR Solé-Casals J. Real-world testing of an artificial intelligence algorithm for the analysis of chest x-rays in primary care settings. Sci Rep. (2024) 14(1):5199. 10.1038/s41598-024-55792-1 38431731 PMC10908781

[6] Sargent W Gibb I. The sensitivity of chest x-ray (CXR) for the detection of significant thoracic injury in children exposed to blast. Injury. (2023) 54(5):1292–6. 10.1016/j.injury.2022.12.001 36539310

[7] Tanabe N Nakagawa H Sakao S Ohno Y Shimizu K Nakamura H. Lung imaging in COPD and asthma. Respir Investig. (2024) 62(6):995–1005. 10.1016/j.resinv.2024.08.014 39213987

[8] Bonaffini PA Stanco F Dulcetta L Poli G Brambilla P Marra P. Chest x-ray at emergency admission and potential association with barotrauma in mechanically ventilated patients: experience from the Italian core of the first pandemic peak. Tomography. (2023) 9(6):2211–21. 10.3390/tomography9060171 38133075 PMC10748272

[9] Naidji MR Elberrichi Z. Automatic detection of COVID-19 from chest x-ray images using EfficientNet-B7 CNN model with channel-wise attention. Int J Com Dig Sys. (2024) 15(1):1443–56. 10.12785/ijcds/1501102

[10] Schalekamp S Van Leeuwen K Calli E Murphy K Rutten M Geurts B. Performance of AI to exclude normal chest radiographs to reduce radiologists' Workload. Eur Radiol. (2024) 34(11):7255–63. 10.1007/s00330-024-10794-5 38758252 PMC11519193

[11] Artsi Y Klang E Collins JD Glicksberg BS Korfiatis P Nadkarni GN. “Large language models in radiology reporting—a systematic review of performance, limitations, and clinical implications”. *Intell-Based Med*. (2025) 12:100287. 10.1101/2025.03.18.25324193

[12] Salehi S Singh Y Horst KK Hathaway QA Erickson BJ. Agentic AI and large language models in radiology: opportunities and hallucination challenges. Bioengineering. (2025) 12(12):1303. 10.3390/bioengineering12121303 41463600 PMC12729288

[13] Dutta N Bose K Syailendra E Chu L Gupta P. Vision-language models in diagnostic imaging: review of technical advances, clinical validation, and practical deployment. Int J Med Inf. (2026) 208:106227. 10.1016/j.ijmedinf.2025.106227 41483727

[14] Ji J Hou Y Chen X Pan Y Xiang Y. Vision-language model for generating textual descriptions from clinical images: model development and validation study. JMIR Form Res. (2024) 8:e32690. 10.2196/32690 38329788 PMC10884898

[15] Kapadnis M Patnaik S Nandy A Ray S Goyal P Sheet D. SERPENT-VLM: self-refining radiology report generation using vision language models. In: Proceedings of the 6th Clinical Natural Language Processing Workshop (Mexico City, Mexico; Association for Computational Linguistics) (2024), 283–91. 10.18653/v1/2024.clinicalnlp-1.24

[16] Li Y Kong C Zhao G Zhao Z. Automatic radiology report generation with deep learning: a comprehensive review of methods and advances. Artif Intell Rev. (2025) 58(11):344. 10.1007/s10462-025-11337-0

[17] Selivanov A Rogov OY Chesakov D Shelmanov A Fedulova I Dylov DV. Medical image captioning via generative pretrained transformers. Sci Rep. (2023) 13(1):4171. 10.1038/s41598-023-31223-5 36914733 PMC10010644

[18] Li Y Yang B Cheng X Zhu Z Li H Zou Y. Unify, align and refine: multi-level semantic alignment for radiology report generation. In: Proceedings of the IEEE/CVF International Conference on Computer Vision. (2023), 2863–74.

[19] Zhang S Han Q Li J Sun Y Qin Y. A medical report generation method integrating teacher–student model and encoder–decoder network. Biomed Signal Process Control. (2024) 94:106251. 10.1016/j.bspc.2024.106251

[20] Chen Z Song Y Chang TH Wan X. Generating radiology reports via memory-driven transformer. In: Proceedings of the 2020 Conference on Empirical Methods in Natural Language Processing (EMNLP) (2020), 1439–49

[21] Wang X Li Y Wang F Wang S Li C Jiang B. “R2gencsr: Retrieving context samples for large language model based x-ray medical report generation,” arXiv preprint arXiv:2408.09743 (2024).

[22] Pereira SC Mendonça AM Campilho A Sousa P Lopes CT. Automated image label extraction from radiology reports — a review. Artif Intell Med. (2024) 149:102814. 10.1016/j.artmed.2024.102814 38462277

[23] Abdaoui H Barki C Dergaa I Tlili K Ceylan Hİ Bragazzi NL. Accurate clinical entity recognition and code mapping of anatomopathological reports using BioClinicalBERT enhanced by retrieval-augmented generation: a hybrid deep learning approach. Bioengineering. (2026) 13(1):30. 10.3390/bioengineering13010030 PMC12838374 41595962

[24] Zeiser FA Da Costa CA De Oliveira Ramos G Maier A Da Rosa Righi R. CheXReport: a transformer-based architecture to generate chest x-ray reports suggestions. Expert Syst Appl. (2024) 255:124644. 10.1016/j.eswa.2024.124644

[25] Hen Z Shen Y Song Y Wan X. Cross-modal memory networks for radiology report generation. In: Proceedings of the 59th Annual Meeting of the Association for Computational Linguistics and the 11th International Joint Conference on Natural Language Processing (Volume 1: Long Papers). Online: Association for Computational Linguistics (2021). p. 5904–14. 10.18653/v1/2021.acl-long.459

[26] Edirisinghe D Nimalsiri W Hennayake M Meedeniya D Lim G. Chest x-ray report generation using abnormality guided vision language model. IEEE Access. (2025) 13:157651–73. 10.1109/ACCESS.2025.3606961

[27] Viana Vargas T Pedrini H Santanchè. LLM-Driven Chest x-ray report generation with a modular, reduced-size architecture. In: Brazilian Conference on Intelligent Systems (Springer) (2024), 199–211

[28] Yuan H. “Natural language processing for chest X-ray reports in the transformer era: bERT-like encoders for comprehension and GPT-like decoders for generation,” iRADIOLOGY. (2025) 3(4):295–301. 10.1002/ird3.115

[29] Heiman A Zhang X Chen E Kim SE Rajpurkar P. FactCheXcker: mitigating measurement hallucinations in chest X-ray report generation models. In: 2025 IEEE/CVF Conference on Computer Vision and Pattern Recognition (CVPR). Nashville, TN: IEEE (2025). 10.1109/CVPR52734.2025.02867

[30] Abo El-Enen M Saad S Nazmy T. A survey on retrieval-augmentation generation (RAG) models for healthcare applications. Neural Comput Appl. (2025) 37(33):28191–267. 10.1007/s00521-025-11666-9

[31] Jiang Y Chen J Yang D Li M Wang S Wu T. CoMT: chain-of-medical-thought reduces hallucination in medical report generation. In: ICASSP 2025 - 2025 IEEE International Conference on Acoustics, Speech and Signal Processing (ICASSP). IEEE (2025). 10.1109/ICASSP49660.2025.10889152

[32] Chen J Huang X Jiang M Li Y Zou Z Qian D. Graph-Driven medical report generation with adaptive knowledge distillation. Appl Sci. (2025) 15(20):10974.

[33] Sun S Su Z Meizhou J Feng Y Hu Q Luo J. Optimizing medical image report generation through a discrete diffusion framework. J Supercomput. (2025) 81(5):637. 10.1007/s11227-025-07111-2

[34] Vaidya SS Palani G Ramesh S Balasubramanian V Selvam M Srinivasaraja G. MedPAO: a protocol-driven agent for structuring medical reports. In: International Workshop on Agentic AI for Medicine. Springer (2025). p. 33–45.

[35] Wang P Ye S Naseem U Kim J. “MRGAgents: A Multi-Agent Framework for Improved Medical Report Generation with Med-LVLMs,” arXiv preprint arXiv:2505.18530 (2025).

[36] Loshchilov I Hutter F. “Decoupled weight decay regularization,” arXiv preprint arXiv:1711.05101 (2017).

[37] Zhang T Kishore V Wu F Weinberger KQ Artzi Y. “Bertscore: Evaluating text generation with bert,” arXiv preprint arXiv:1904.09675 (2019).

[38] Papineni K Roukos S Ward T Zhu WJ. Bleu: a method for automatic evaluation of machine translation. In: Proceedings of the 40th Annual Meeting of the Association for Computational Linguistics (Philadelphia, Pennsylvania, USA: Association for Computational Linguistics) (2002), 311–8. 10.3115/1073083.1073135

[39] Lin CY. ROUGE: a package for automatic evaluation of summaries. In: Text Summarization Branches out (Barcelona, Spain: Association for Computational Linguistics) (2004), 74–81

[40] Banerjee S Lavie A. METEOR: an automatic metric for MT evaluation with improved correlation with human judgments. In: Proceedings of the ACL Workshop on Intrinsic and Extrinsic Evaluation Measures for Machine Translation and/or Summarization (Ann Arbor, Michigan, Association for Computational Linguistics) (2005), 65–72

[41] Irvin J Rajpurkar P Ko M Yu Y Ciurea-Ilcus S Chute C. CheXpert: a large chest radiograph dataset with uncertainty labels and expert comparison. Proceedings of the AAAI Conference on Artificial Intelligence. (2019) 33:590–7. 10.1609/aaai.v33i01.3301590

[42] Jain S Agrawal A Saporta A Truong SQH Duong DN Bui T. RadGraph: extracting clinical entities and relations from radiology reports. arXiv:2106.14463 [cs.CL] (2021). 10.48550/arXiv.2106.14463

[43] Liu G Hsu TMH McDermott M Boag W Weng WH Szolovits P. Clinically accurate chest X-ray report generation. In: Proceedings of the 4th Machine Learning for Healthcare Conference. PMLR (2019) 106:249–69.

[44] Miura Y Zhang Y Tsai E Langlotz C Jurafsky D. Improving factual completeness and consistency of image-to-text radiology report generation. In: Proceedings of the 2021 Conference of the North American Chapter of the Association for Computational Linguistics: Human Language Technologies. (2021), 5288–304

[45] Zhao W Wu C Zhang X Zhang Y Wang Y Xie W. Ratescore: a metric for radiology report generation. In: Proceedings of the 2024 Conference on Empirical Methods in Natural Language Processing. (2024), 15004–19

[46] Zhang X Zhou HY Yang X Banerjee O Acosta JN Miller J. ReXrank: a public leaderboard for AI-powered radiology report generation. arXiv:2411.15122 [cs.CV] (2024). 10.48550/arXiv.2411.15122

[47] Yu F Endo M Krishnan R Pan I Tsai A Reis EP. Evaluating progress in automatic chest X-ray radiology report generation. Patterns. (2023) 4(9):100802. 10.1016/j.patter.2023.100802 37720336 PMC10499844

[48] Chen Z Shen Y Song Y Wan X. Cross-modal memory networks for radiology report generation. arXiv:2204.13258 [cs.CL] (2022). 10.48550/arXiv.2204.13258

[49] Yi Z Xiao T Albert MV. “A multimodal multi-agent framework for radiology report generation,” arXiv preprint arXiv:2505.09787. (2025).

[50] Xia P Zhu K Li H Zhu H Li Y Li G. Rule: reliable multimodal RAG for factuality in medical vision language models. In: Proceedings of the 2024 Conference on Empirical Methods in Natural Language Processing. 2024. p. 1081–93.

[51] Han S Xia P Zhang R Sun T Li Y Zhu H. MDocAgent: a multi-modal multi-agent framework for document understanding. arXiv:2503.13964 [cs.CL] (2025). Available online at: https://arxiv.org/abs/2503.13964 (Accessed July 20, 2026).

[52] Qi T Bu S Xiang Y Dai Z. “EviAgent: Evidence-Driven Agent for Radiology Report Generation,” arXiv preprint arXiv:2603.13956 (2026).

[53] Wang P Ye S Naseem U Kim J. Mrgagents: a multi-agent framework for improved medical report generation with med-lvlms. In: 2025 International Conference on Digital Image Computing: Techniques and Applications (DICTA) (IEEE) (2025), 1–7.

[54] Johnson AEW Pollard TJ Berkowitz SJ Greenbaum NR Lungren MP Deng CY. MIMIC-CXR, a de-identified publicly available database of chest radiographs with free-text reports. Sci Data*.* (2019) 6(1):317. 10.1038/s41597-019-0322-0 31831740 PMC6908718

[55] Chambon P Delbrouck JB Sounack T Huang SC Chen Z Varma M. CheXpert plus: augmenting a large chest X-ray dataset with text radiology reports, patient demographics and additional image formats. arXiv:2405.19538 [cs.CL] (2024). 10.48550/arXiv.2405.19538

