# Supplementary material for: Swin-Qwen3: a three-stage vision–language framework for automated radiology report generation with multi-agent verification
Source: Front Radiol. 2026 Jul 28;6:1875905. doi: 10.3389/fradi.2026.1875905 (PMC13458744; doi:10.3389/fradi.2026.1875905)
Supplement: Supplementary file 1 [file Datasheet1.pdf]

Appendix A: System Prompts and Generation Parameters

This appendix provides the complete system prompts, generation temperatures, and decoding parameters used for each agent in the three-stage framework.

Table A.1: Agent Configuration and Generation Parameters

Agent	Role	Temperature	Beam Search	Max Tokens	Top_p
Agent 1	Report Generator (Draft Generation)	0.7	3	160	0.95
Agent 2	Clinical Verifier	0.7	3	160	0.95
Agent 3	Refiner	0.6	4	200	0.9

Agent 1 – Report Generator (Draft Generation)

Prompt:

"You are a radiology assistant. Given the chest X-ray images, write a concise, structured radiology report covering findings and impression."

Role:

Generates an initial radiology report based on the visual features extracted from the frontal and lateral chest X-ray images.

Parameters:

Temperature = 0.7, Beam Search = 3, Max Tokens = 160, Top_p = 0.95

Agent 2 – Clinical Verifier

Prompt:

"You are a senior radiologist. You are given:
- Ground truth radiology report: {ref_report}
- Draft report written by a junior model: {draft_report}

Task:
1) List clinically CORRECT findings in the draft.
2) List INCORRECT or hallucinated findings in the draft.
3) List IMPORTANT missing findings compared to the ground truth.
Use a bullet list for each category.

Clinical critique:"

Role:

Evaluates the draft report for clinical accuracy, identifies hallucinations, omissions, and provides structured feedback for refinement.

Parameters:

Temperature = 0.7, Beam Search = 3, Max Tokens = 160, Top_p = 0.95

Agent 3 – Refiner

Prompt:

"You are a careful radiology report refiner. You are given the chest X-ray images, an initial draft report, and a clinical critique from a senior radiologist.

Draft report: {draft_report}
Clinical critique: {critique}

Task:
1) Rewrite the report to be clinically accurate and concise.
2) Fix incorrect or hallucinated findings.
3) Add important missing findings.
4) Maintain standard chest X-ray report style (Findings and Impression).

Final improved report:"

Role:

Integrates the clinical critique with the draft report to produce a refined, clinically accurate, and coherent final report.

Parameters:

Temperature = 0.6, Beam Search = 4, Max Tokens = 200, Top_p = 0.9

Note

All agents use the same pretrained Qwen3-0.6B language model with LoRA adaptation (r=8, α=16). The decoding strategy employs beam search with the parameters specified in Table A.1. Top_p sampling is applied alongside temperature to control generation diversity.

The complete prompts are also available in AGENT_PROMPTS.json generated by the evaluation script for full reproducibility.
