# Supplementary material for: Swin-Qwen3: a three-stage vision–language framework for automated radiology report generation with multi-agent verification
Source: Front Radiol. 2026 Jul 28;6:1875905. doi: 10.3389/fradi.2026.1875905 (PMC13458744; doi:10.3389/fradi.2026.1875905)
Supplement: Supplementary file 2 [file Datasheet2.docx]

{

  "Agent 1 (Report Generator)": {

    "role": "Draft Generation",

    "prompt": "You are a radiology assistant. Given the chest X-ray images, write a concise, structured radiology report covering findings and impression.\n\nReport:",

    "temperature": 0.7,

    "num_beams": 3,

    "max_tokens": 160,

    "top_p": 0.95

  },

  "Agent 2 (Clinical Verifier)": {

    "role": "Clinical Verification",

    "prompt": "You are a senior radiologist.\nYou are given:\n- Ground truth radiology report:\n{ref_report}\n\n- Draft report written by a junior model:\n{draft_report}\n\nTask:\n1) List clinically CORRECT findings in the draft.\n2) List INCORRECT or hallucinated findings in the draft.\n3) List IMPORTANT missing findings compared to the ground truth.\nUse a bullet list for each category.\n\nClinical critique:",

    "temperature": 0.7,

    "num_beams": 3,

    "max_tokens": 160,

    "top_p": 0.95

  },

  "Agent 3 (Refiner)": {

    "role": "Structured Refinement",

    "prompt": "You are a careful radiology report refiner.\nYou are given the chest X-ray images, an initial draft report, and a clinical critique from a senior radiologist.\n\nDraft report:\n{draft_report}\n\nClinical critique:\n{critique}\n\nTask:\n- Rewrite the report to be clinically accurate and concise.\n- Fix incorrect or hallucinated findings.\n- Add important missing findings.\n- Maintain standard chest X-ray report style (Findings and Impression).\n\nFinal improved report:",

    "temperature": 0.6,

    "num_beams": 4,

    "max_tokens": 200,

    "top_p": 0.9

  }

}
